#  The NIST Vacuum Double-Crystal Spectrometer: A Tool for SI-Traceable Measurement of X-Ray Emission Spectra

**DOI:** 10.6028/jres.126.049

**Published:** 2022-03-09

**Authors:** Csilla I. Szabo, James P. Cline, Albert Henins, Lawrence T. Hudson, Marcus H. Mendenhall

**Affiliations:** 1 National Institute of Standards and Technology, Gaithersburg, MD 20899, USA; 2 Theiss Research, La Jolla, CA 92037, USA

**Keywords:** double-crystal spectrometer, wavelength standards, x-ray spectroscopy, x-ray transition energies

## Abstract

The NIST Vacuum Double-Crystal Spectrometer (VDCS) has been modernized and is now capable of recording reference-free wavelength-dispersive spectra in the 2 keV to 12 keV x-ray energy range. The VDCS employs crystals in which the lattice spacings are traceable to the definition of the meter through x-ray optical interferometry with a relative uncertainty ﹤10ˉ⁸. VDCS wavelength determination relies upon precision angle difference measurements for which the encoders of the rotation stages have been calibrated using the circle closure method for accurate, absolute angle measurement. The new vacuum-compatible area detector allows quantification of the aberration functions contributing to the observed line shape and in situ alignment of the crystal optics. This latter procedure is augmented with the use of a thin lamella as the first crystal. With these new techniques, x-ray spectra are registered with the VDCS on an absolute energy scale with a relative uncertainty of 10ˉ⁶.

## 1 Background

The Vacuum Double- Crystal Spectrometer (VDCS) was first implemented at the National Institute of Standards and Technology (NIST) in the 1960s [1] when there was general interest to create lists of the characteristic x-ray radiation lines of the elements [2]. The first realization of combined x-ray optical interferometry by Deslattes and Henins [3] made it possible to make measurements of the lattice spacings of silicon crystals traceable to the International System of Units (SI). This and the development of lattice spacing comparison techniques [4] meant the extension of SI traceability to x-ray wavelengths and, through the *hc/e* conversion constant (where *h* is the Planck constant, *c* is the speed of light, and *e* is the electron charge), to the energy scale. As of May 20, 2019, the conversion constants of *h*, *c*, and *e* are exactly defined, and this ratio is approximately 1 239.841 984 332 eV nm [5]. Results from the VDCS now offer a direct traceability that can be followed from the SI definition of the meter to the measured x-ray energy scales [6].

The first comprehensive list of x-ray transition energies, including SI traceable values, was published in 2003 [7] and was adapted as a Standard Reference Database (SRD), SRD 128 [8], at NIST. This database summarizes work and measurement efforts performed over the course of several decades [9]. While the published *K* and *L* x-ray transitions include only a limited number of SI-traceable measurements, many precise relative measurements could be placed on a common traceable energy scale (see Tables I and II in Ref. [7]). Another strength of this work was the publication of calculated theoretical values for each listed x-ray transition, which allowed for the observation of extreme discrepancies. As the authors admitted, this database is “far from perfect,” but it does provide a view of the state of the art and its development for the measurement of transition energies. The database includes measurements that date back to the beginning of the twentieth century shortly after the discovery of x-ray diffraction. Today, the same x-ray diffraction methods, utilizing traceable crystals embedded in fully automated and temperature-controlled environments, can provide improvement for even the best previously measured spectra, *e.g*., the *K* spectrum of copper [10].

The data in SRD 128 consist of “lines” or the position in the emission spectra of maximum intensity. However, the characteristic x-ray emission spectra are in fact complicated profiles. A spectrum is composed of multiple transitions, which produce complex asymmetric profile shapes. In many cases, the spectrum is highly dependent on the chemical state of the target, creating chemical shifts that also alter the apparent emission spectra. In principle, an analysis of these profiles requires the summation of every contribution attributable to each transition. However, analysis of these spectra considering all transitions is not undertaken; instead, multiple analytical profile shape functions, such as Lorentzians, are used to fit the observed profiles. In the case of the Cu *Kα* emission spectrum, typically four Lorentzian profiles are used to fit the observation, but these have no fundamental correlation to the underlying transitions [11, 12].

Ideally, the production of reference x-ray data would rely on SI-traceable, high-resolution, and high-sensitivity methods to provide detailed profile shapes of both low- and high-intensity peaks over a large energy range. The wavelength-dispersive double-crystal method provides high-resolution (relative uncertainty, Δ*E*/*E* ≈ 10^−6^) spectra of intense transitions above energies of 2000 eV. Energy-dispersive detectors have offered high data-acquisition rates but have traditionally suffered from low resolution. Nevertheless, newer transition edge sensor (TES) arrays can offer high sensitivity and good resolution of Δ*E*/*E* ≈ 10^−4^ across a wide energy range [13, 14]. These detectors, however, do not have an inherent energy calibration and must therefore rely on tabulated reference lines in the database. Reliance on the single point values in SRD 128, rather than actual profiles, leads to a loss of accuracy in energy calibration. The VDCS and TES-based instruments are complementary in the production of reference data [15, 16]; the wavelength-dispersive double-crystal method provides SI-traceable, low-bandwidth, high-resolution spectra of intense transitions, while the TES spectrometers can register low-intensity transitions at a relatively good resolution across a wide energy range.

There are many other groups around the globe who are using or developing the double-crystal spectrometer (DCS) method for various applications. One example is the use of a DCS for the investigation of x-ray transitions in highly charged ions (HCIs) at the Laboratoire Kastler Brossel (LKB) in Paris [17]. The Paris DCS provides SI-traceable measurements of narrow, well-described x-ray transitions in HCIs that test quantum electrodynamics (QED) theories [18-20]. A group in Kyoto, Japan, uses the dispersive mode of the DCS [21] to determine line shapes and satellite lines with x-ray tube excitation [22-24] or using synchrotron radiation [25]. A group in Lisbon, Portugal, is developing their own DCS in vacuum to support their substantial theoretical work [26-28] with experimental data.

In this paper, we demonstrate the performance of the modernized NIST VDCS by the measurement of the Cu *Kα* spectrum. Due to its ease of production and transition energy, copper is well studied [29-34] and is an important reference for calibrations in x-ray science, *e.g.*, x-ray fluorescence (XRF) and x-ray emission spectroscopy (XES) studies [15, 16]. In addition, the shape of the Cu K*α* spectrum is important for the proper analysis of x-ray diffraction (XRD) spectra for powder diffraction studies [10, 35].

## 2 The NIST Vacuum Double-Crystal Spectrometer

### 2.1 History of the NIST VDCS

The VDCS [1] was first initiated at NIST by Richard D. Deslattes in the early 1960s. The construction of a “vacuum instrument” was undertaken to extend the measurement range and capabilities of double-crystal instruments operating in air [36]. In the early stages of its existence, the current-stabilized [37] x-ray source [38] was equipped with the capability to fluoresce gaseous targets [39]. Various measurements with argon and potassium chloride [40, 41] were performed, and the measurements were subsequently extended to emission spectra from chlorinated hydrocarbons, fluorocarbon molecular gases [42], and sulfur hexafluoride [43, 44]. Up until the completion of this work, the VDCS was operating with a gas-filled proportional counter [39]. The NIST VDCS was equipped to perform measurements in the soft x-ray region and on molecules [45]. It was also used to measure the *L* series of germanium [46], the *M* series of xenon [47], and the *K* spectrum of argon [48].

In the 1970s, LaVilla published numerous results from precision studies of various elements, including *M* emission spectra of gadolinium and ytterbium oxides [49], and emission and absorption spectra of oxygen and carbon [50]. He also studied *Kβ* emission and *K* absorption spectra of sulfur [51], *L* gamma spectra of tellurium [52] and tin and iodine [53], and the copper *Kβ* spectrum [54].

In the early 1980s, the VDCS provided reference data in the form of the argon and potassium *Kα* lines for the measurements of the 1*s* Lamb shift in hydrogen-like chlorine [55] and argon [56], respectively. In 1985, various measurements performed with the VDCS and other instruments at NIST were summarized and combined with theory in a review work by Deslattes [9]. Later on, papers were published by Ohno and LaVilla based on measurements on the *L* gamma emission spectra of xenon [57], rare earth elements [58], and barium oxide [59].

In the 1990s, the *L* emission spectra of xenon were studied with the VDCS [60, 61], and precision measurements were performed to produce x-ray energy profiles of Mg and Al *K* transitions [62]. Later, Mooney performed a great number of precision measurements on various *L* and *K* transitions, and those results were included as reference 3 of Table V in the review article about the x-ray transitions in 2003 [7]. These measurements included *K* transitions from Si, S, Cl, Ga, As, Se, and Kr and *L* transitions from Kr, Zr, Nd, Sm, Ho, Er, and Tm, among others.

Modification to the originally described [1] spectrometer included the upgrade of the vacuum system from diffusion pumps to turbomolecular high-vacuum pumps. Also see Sec. 2.4 for more details. Another major alteration to the original design was the modification of the axial drives and the installation of angle encoders on the axes that replaced the originally installed tangent arms and other components. This modification occurred in the early 1990s. Section 2.3 provides the description of the current spectrometer assembly after an overall upgrade and maintenance of almost all components of the instrument.

### 2.2 Principle of Operation

Double-crystal spectrometers have been used for precision x-ray measurements since the early 1920s [63]. For a description of the development of the double-crystal technique, see the Introduction of Ref. [17]. The operation has been described many times in articles and textbooks [17, 64]. Systematic corrections, *e.g*., axial divergence, were first introduced by Williams [65] and further explored by Bearden [66], while Mendenhall [10] described the first use of a two-dimensional detector to address this and other systematics. Here, we give a short summary of the operation.

**Fig. 1:**
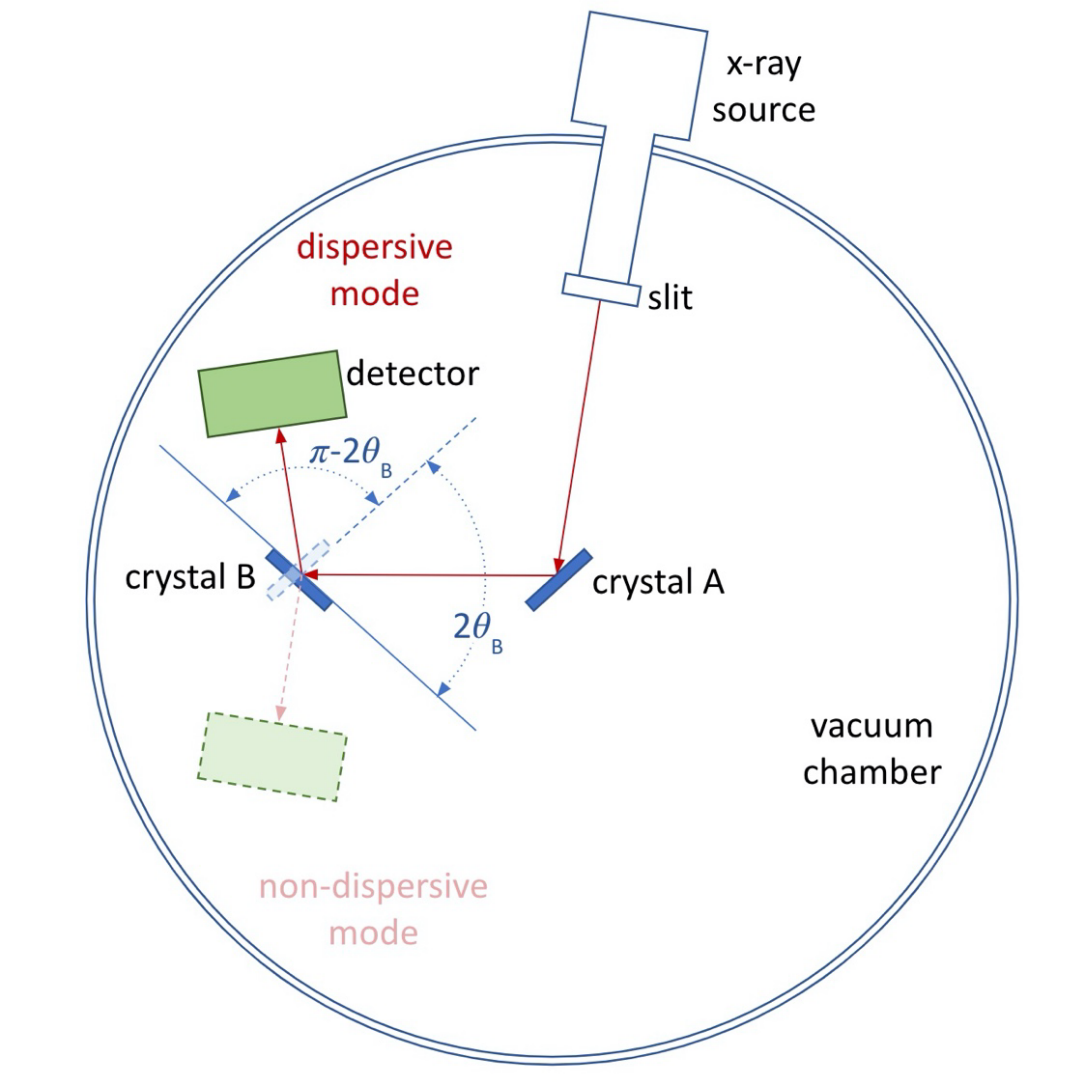
Setup and operation principle of the NIST VDCS.

Double-crystal spectrometers employ an x-ray source, two crystals as diffraction elements, and an x-ray detector. In the case of the NIST VDCS, the source is attached to the vacuum chamber, which can be rotated with respect to the fixed platform holding the x-ray optics and detector. The first crystal and the source are set to a position such that the scattered radiation from the first crystal is directed toward the second crystal when the diffraction condition is satisfied, *viz*: nλ=2dsinθ (Bragg’s law), where *λ* is the desired x-ray wavelength,^1^1 X-ray wavelength and energy can be used interchangeably in the context of this report because their values are precisely related through the universal constant *hc*/*e*, which has the value of 1 239.841 984 332 eV nm, as described in the text.
*θ* is the diffraction angle, *d* is the crystal lattice spacing, and *n* is the diffraction order. The second crystal can be set in two different orientations to the first, non-dispersive and dispersive, where signal can be registered on the detector while scanning the second crystal. Figure 1 illustrates the concept of operation of the NIST VDCS.

In the non-dispersive mode, crystals A and B are positioned parallel to one another to satisfy Bragg’s law for an x-ray wavelength region of interest. The non-dispersive geometry leads to diffraction of the entire range of wavelengths over a very narrow angular range approximating the Darwin width of the two diffraction crystals. With the VDCS, crystal B is scanned by rotating it around an axis perpendicular to the diffraction plane, while crystal A is held stationary. The non-dispersive diffraction peak so generated is the autocorrelation of the crystal A and crystal B single-crystal rocking curves. This profile constitutes the intrinsic instrumental resolution of a VDCS. The position of the center of this curve, *θ*_non-disp_, is used as a reference point to measure the angle differences from the angular measurements, *θ*_disp_, during the dispersive spectral scan.

In the dispersive mode, crystal B and the detector are positioned in a geometry where the high-resolution diffracted x-ray spectrum can be registered. Here, crystal B is scanned again in fine steps to record a high-resolution, dispersed x-ray spectrum. The angle difference measured between the non-dispersive peak angle, *θ*_non-disp_, and the scanned angles in the dispersive mode, *θ*_disp_, is related to the Bragg angle, *θ*_B_, through Eq. (1).

${2\theta }_{\text{B}}=\left[180-\left(\theta_{\text{non-disp}}-\theta_{\text{disp}}\right)\right]$ (1)

where *θ*_disp_ is the angle measured in the dispersive mode for any part of the dispersed spectrum. In a double-crystal spectrometer instrument, both angles, *θ*_non-disp_ and *θ*_disp_, are directly measured by high-precision angle encoders relative to a previously determined zero crystal position. Hence, the double-crystal method has the metrological advantage of employing a difference measurement with no need for an external wavelength reference. The Bragg equation provides the link between the measured Bragg angle (*θ*_B_) and the x-ray wavelength (*λ*) through the SI-traceable lattice spacing (*d*), which has been determined for silicon by x-ray optical interferometry. A detailed description of the traceability chain can be found in Ref. [6].

### 2.3 VDCS Overview

**Fig. 2:**
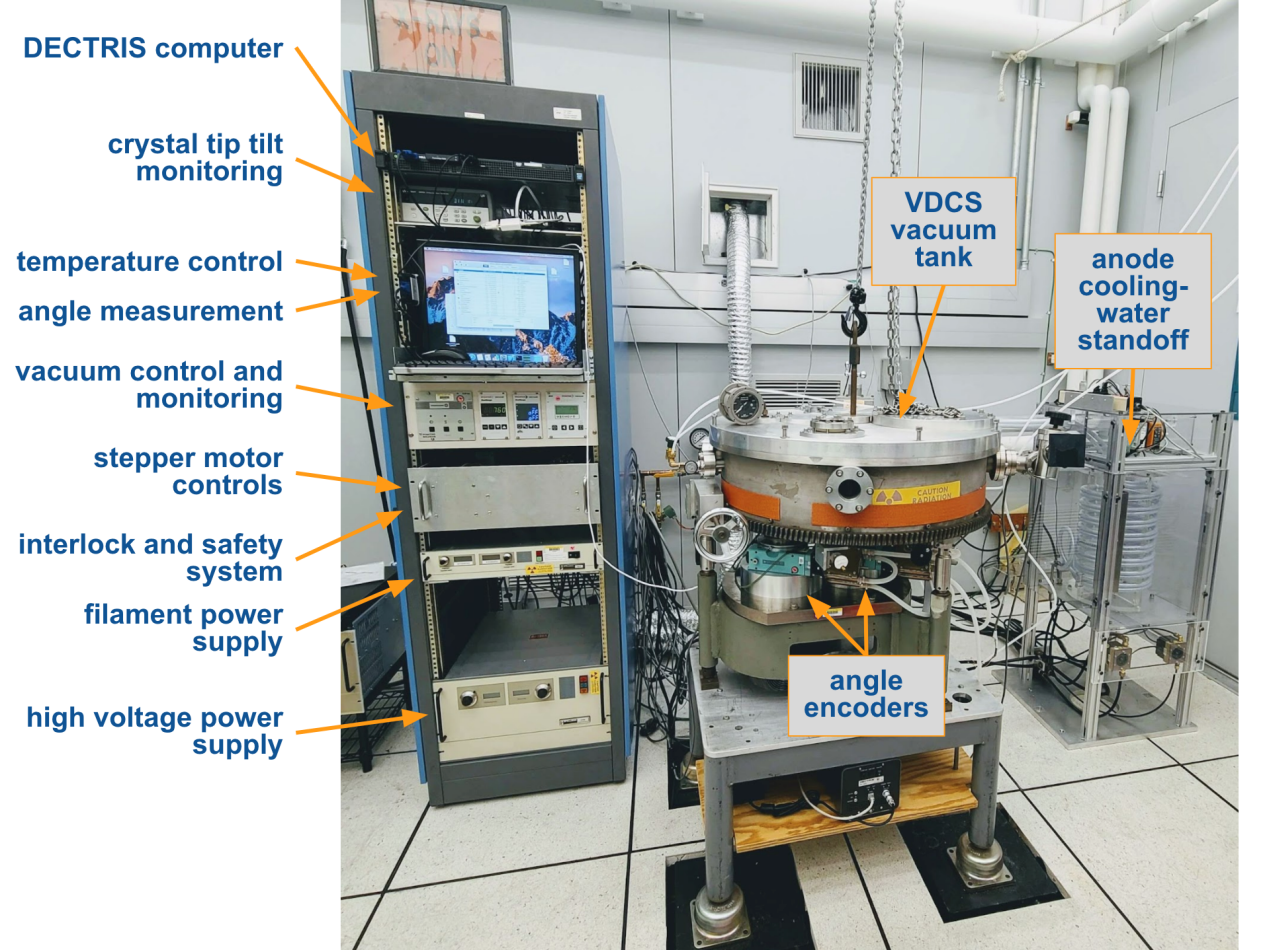
Overview of the VDCS laboratory with control electronics on the left, the VDCS vacuum chamber in the middle, and the high-voltage anode cooling-water standoff on the right.

The VDCS instrument is in an environmentally controlled laboratory space in one of the subterranean buildings of the NIST Advanced Measurement Laboratory. In the following sections, all major instrument components and the laboratory environment will be described. A view of the NIST VDCS laboratory is shown in Fig. 2. The control electronics are seen on the left, the large circular vacuum tank of the spectrometer is located in the middle, and the anode cooling-water standoff is on the right. The demountable x-ray source is mounted on the rear of the tank and is not visible in the picture. Most of the control electronics were replaced during the modernization of the instrument.

From top to bottom of the control rack, under the rack-mountable computer, the first shelf includes the crystal tip-tilt control. The second shelf stores the temperature measurement and the angle encoder control units behind the computer screen. Under these, there are the vacuum control and pressure measurement devices, which will be explained further in the next section. The aluminum drawer includes all the stepper motor controls and support electronics for the interlock and safety system. The bottom two power supplies service the x-ray generator, as described in Sec. 2.5.

Each of the diffraction crystals is mounted on a vertical-axis goniometer equipped with a high-precision rotation stage (Huber 410 on both axes)^2^2 Certain commercial equipment, instruments, or materials are identified in this paper to foster understanding. Such identification does not imply recommendation or endorsement by the National Institute of Standards and Technology, nor does it imply that the materials or equipment identified are necessarily the best available for the purpose. and encoders (A axis: Canon X-1, B axis: Heidenhain RON 905). These were installed in the 1990s, modifying the original design [1]. The rotation stages and encoders are mounted under the vacuum tank of the instrument in air. The rotation stage of axis B can be seen as the green component in Fig. 2 under the vacuum tank. The encoders are housed beneath the rotation stages. From both rotation stages, a 25.4 mm diameter hardened steel shaft extends down into its encoder and upward, through dynamic vacuum seals, into the vacuum chamber, providing the axes for crystals A and B to be mounted. The overall physical setup of the spectrometer vacuum tank and the axis assemblies has not been changed from that of the original design and can be observed on the bottom half of Fig. 1 in Ref. [1]. The vacuum seals decouple the precision rotation system of the axes from possible distortions due to venting and evacuation of the tank.

The base plate of the vacuum chamber rests on kinematic mounts on the base support. The three legs of the vacuum tank were extended to make room for the current precision goniometers and encoders. The rotation stages and encoders are placed on a steel plate that can be leveled; the electronics for the x-ray detector are shelved beneath this assembly. The entire assembly is sitting on a vibration isolation platform; the black support blocks visible in Fig. 2 aligned with the flooring of the laboratory are meter-long steel columns that rest on a 23000 kg (23 ton) concrete pier in the lower level.

The goniometers are driven by stepper motors with harmonic drive gear reducers. The motors are water cooled to reduce unwanted heat conduction to the vacuum tank during operation. The encoders are read out by a Heidenhain EIB741 interpolation interface. Section 2.7 will provide a short description of the verification and calibration procedure for the encoders. The instrument is controlled via LabVIEW; communication with the various interfaces is largely through a local network.

**Fig. 3:**
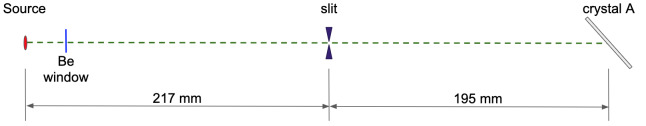
Line of sight from the x-ray source to crystal A.

The x-ray source is attached to the wall of the main vacuum tank that can be rotated. As seen on the sketch of Fig. 1, crystal A is placed on the rotation stage in the center of the cylindrical vacuum chamber, and crystal B is placed on the rotation axis 190 mm away from the crystal A axis. Figure 3 shows the line of sight and measured distances from the x-ray source to crystal A. The horizontal slit provides a 0.2 mm gap between stainless-steel blades; this assists in crystal tilt alignment and axial divergence corrections as discussed below. The two-dimensional (2D) silicon detector face is approximately 101 mm away from the axis of crystal B.

**Fig. 4:**
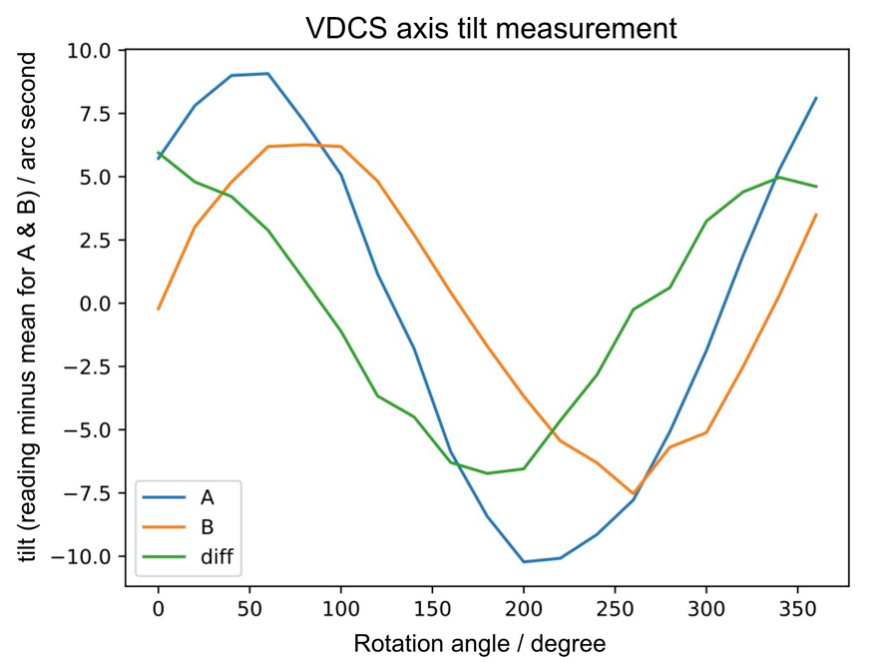
Tilt of axis A and axis B with respect to a parallel vertical axis and their difference. Tilt means instrument reading minus the mean measurement value, for each axis.

The alignment of the spectrometer axes was checked with an LSRP-1 inclinometer from Jewell Instruments. After leveling the spectrometer platform, the inclinometer was attached to the top of the A axis and then to the top of the B axis spindle. Both axes were scanned over a full circle with a stepping routine controlled by LabView, and the inclinometer readings were recorded. The result of the two scans and the difference of the curves are shown in Fig. 4.

The maximum 6 arc second angle difference measured between the two axes is well within the maximum roughly 100 arc second misalignment limit determined from calculations based on the VDCS geometry to maintain a relative uncertainty Δ*E*/*E* close to 10^-6^ at the Cu *Kα* x-ray energy. For more details, see Sec. 3.2.

### 2.4 Vacuum System

The vacuum system includes the tank, nominally 70 cm in diameter, wherein the crystals and the detector reside, and a second system for the demountable x-ray source. Both the tank and the source employ turbo-molecular pumps that are roughed by a common scroll pump. A high vacuum (<1.3^−5^ Pa ≈ 10^−7^ Torr) ensures the operation of the filaments of the x-ray source, and a lower vacuum helps the transmission of the x-rays without absorption inside the tank. As described earlier, the base plate of the tank rests on the locator posts and is stationary, providing support for an ~70 cm diameter bearing that in turn supports the outer wall of the vacuum tank. A <70 cm O-ring provides a dynamic seal between the base plate and the rotatable portion of the vacuum chamber, which includes the source. The crystal rotation assemblies attached to the base plate also include dynamic seals. These shaft seals are standard commercial oil seals from which the springs have been removed to further reduce friction and to make them more flexible for the alignment of the rotation assemblies. The base plate has been machined to include flanges for attachment of the vacuum pump and feedthroughs for water cooling, thermistors, and control and sensing electronics related to crystal tilt adjustment.

The tank wall also has apertures for flanges to support the source, vacuum gauges, and observation windows. The lid of the tank consists of an aluminum plate with some additional ports and a hoist point for the crane that can lift and remove either the lid or the lid and tank assembly from the base plate. The vacuum gauges attached to the tank include a simple manometer capable of displaying pressures up to 100 kPa. There are two full-range vacuum gauges installed on the system: one monitors the tank pressure, and the other one is for the source vacuum. A typical reading for these gauges would be ≈ 6.67 × 10^−3^ Pa (≈ 5 × 10^−5^ Torr) for the tank and ≈ 2.67 × 10^−6^ Pa (≈ 2 × 10^−8^ Torr) for the source, with the source off. When the source is operational, readings for the source pressure are up to two orders of magnitude higher. The pressures are monitored for safe operation of the source and the detector; readings are fed into a LabView routine on the control computer.

### 2.5 X-Ray Source

**Fig. 5:**
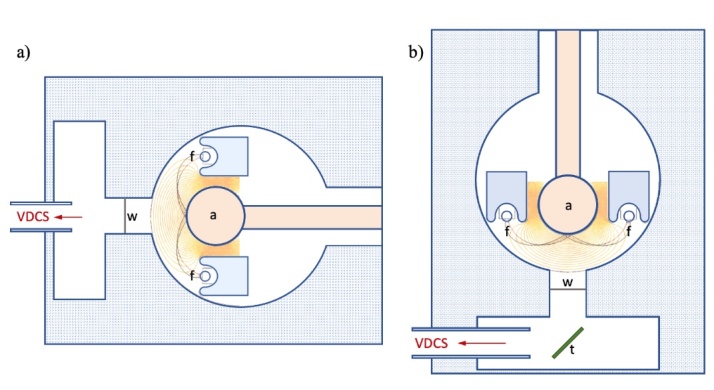
Schematic of the x-ray tube in (a) direct excitation mode and (b) indirect or fluorescence mode. Electrons from the directly heated filaments, *f*, are accelerated to the water-cooled anode *a*, forming a broad focal spot opposite the exit window *w*. A fluorescence target is labeled *t*.

The x rays under study are generated by a demountable x-ray source designed and built in the late 1960s [38]. The advantages of this unique x-ray source include versatility and high power. It can be set up in two different modes: direct excitation mode, exciting the anode with electrons, or indirect excitation of a secondary target by the x rays from the primary target in the x-ray fluorescence mode. The source design also permits the choice of a variety of anode and fluorescence target materials and combinations. For example, a chromium anode [*E*(*Kα*_1_) ≈ 5415 eV, *E*(*Kα*_2_) ≈ 5406 eV] is typically used to fluoresce a scandium foil target [*E*(*Kα*_1_) ≈ 4091 eV, *E*(*Kα*_2_) ≈ 4086 eV] to obtain optimum source brightness. Figure 5 shows schematics of the x-ray source in its two modes. Figure 5(a) shows the direct excitation mode, where the VDCS observes the x-ray source spot directly created on the surface of the water-cooled anode. The electrons generated in both filaments, *f*, are accelerated toward the anode *a*. Figure 5(b) shows the fluorescence mode, where the x rays generated on the surface of the anode will excite a secondary target *t*. The target is usually a metal foil placed at a 45° angle to the axis of the source. When operated in fluorescence mode, there exists no Be window between the actual source of the radiation, the fluorescent target, and the spectrometer, ensuring an undistorted spectrum measurement. The Be window (marked with *w* on Fig. 5) is 0.025 mm thick, which is optimal for low energies and allows nearly 100% transmission at 8 keV x-ray energy. Another advantage of the demountable x-ray source is the relatively high power that can be reached (≈1 kW). This is possible due to the relatively large spot size, as the electrons are not focused to one point on the anode but are spread out over an area of about 8 mm by 10 mm. Also, the filaments are operated at a relatively high current, up to 16.2 A. In the original 1960s design, this high current required the anode to be charged positively while the filaments were held at ground. We retained this approach with our current x-ray generators.

The experiments described in this report were performed in direct excitation mode. The anode was a copper tube bent to a U shape without any coating. The high voltage (HV) applied to the anode was 20 kV, with a maximum emission current of 100 mA. The two tungsten filaments were connected in series heated by a maximum 16.2 A current during the measurements. The water-cooled anode was at positive high voltage, which made it necessary to extend the length of the water cooling lines to reduce leakage current. To allow the cooling water inside the in and out branches to reach HV from ground, plastic tubing was used approximately 7 m in length each. The pair of tubing was coiled in an insulated plastic structure shown in the right of Fig. 2 as the anode cooling-water standoff.

One of the most important requirements for the x-ray source of the VDCS is stability. Keeping emission currents constant at ± 0.1% has been a challenge from the conception of the instrument. This level of stability is an essential requirement for precision measurements with the VDCS, where angle scans can require many hours to obtain satisfactory counting statistics. Given this requirement and that the anode must be positively charged, the generators have consisted of two subcomponents. A high-power (6 kW) and high-voltage (30 kV) power supply (HVPS) to energize the anode of the x-ray source to positive polarity and a high-current (20 A, 20 V) filament power supply (FPS) for electron generation. The connection between the two power supplies, which is essential for the safe and stable operation of the x-ray source, is facilitated through a programmable logic device (PLD). The PLD regulates the ramping of the high voltage on the anode and the current on the filament of the x-ray source in a manner that is opaque to the user.

In the system currently being commissioned, this built-in PLD is to be replaced with high-level programming via LabView to provide more flexibility, *viz.* adjustment of voltage and current ramp-up times and feedback on time constants regulating beam current. This synchronization software integrates the operation of the HVPS and FPS and provides for continuous monitoring of all variables and regulation of the filament current through a proportional-integral-derivative algorithm. The new system will also feature full integration with the interlock system of the VDCS, including the cooling and vacuum systems.

### 2.6 Temperature Control

The laboratory space within which the VDCS instrument is located is controlled at ambient temperature to ± 0.1 °C. The anode of the x-ray source and the turbo pumps are directly cooled by the facility chilled water in the laboratory. There is a low-pressure water chiller set to ambient temperature operating outside the temperature-controlled laboratory that is used to cool the various components of the machine and avoid temperature load on the vacuum chamber. This assures a constant temperature of the crystals, the 2D x-ray detector in the vacuum chamber, and the stepper motors of the rotation stages. The operation of the power supplies of the x-ray source (x-ray generator) produces significant heat that is extracted to a vent through air ducts.

Temperature is monitored in various places throughout the laboratory with temperature probes (5611A, 10 kΩ thermistors) connected to a Hart/Fluke BlackStack system (model 1560). The thermistor probes are calibrated with a NIST-traceable procedure to better than 0.010 °C (*k* = 2) uncertainties. There are thermistor probes attached to the base of both crystals and to the vacuum base plate of the tank. The temperature readings from the crystals are used for corrections of the crystal lattice spacing. Temperature excursions also contribute to the uncertainties of the final spectral measurements. Additional thermistors are placed on the goniometer base plate and on the laboratory wall to monitor the overall outside temperature. To gain information about the efficiency of water cooling, the incoming and outgoing facility water temperatures are also monitored for the anode and the turbo molecular pumps. The facility includes a computer for continuous monitoring of these parameters independent of the data collection routines.

As described, the temperatures of the crystals are recorded during the measurements, and an average temperature is calculated and used to determine the exact value of the lattice spacing for the analysis. The absolute value of the fluctuation of the crystal temperature during one measurement is not larger than 0.2 °C.

### 2.7 Encoder Calibration

Precise angle measurement is the primary measurand of the double-crystal technique. In particular, it is the difference angle from the centroid/peak of the symmetric non-dispersive scan of crystal B relative to any point on the dispersive scan of crystal B. To obtain the desired final uncertainty on x-ray transition energies, the encoders need to function at a smaller uncertainty than that specified by the encoder manufacturer. This is achieved through a calibration curve generated using a circle closure methodology. The encoder manufacturer will specify installation tolerances that, if observed, will result in the encoder operating within the manufacturer’s specifications. However, the only means to test this is to calibrate the encoder. Furthermore, the “calibration curve” that the encoder will exhibit will invariably be a function of the slight misalignments in its installation, and it will be temporally stable. It is the case that the disassembly and reassembly of the VDCS instrument and its axes can lead to changes to the coupling of the axes to the encoder that might modify the calibration function [67]. To be clear, our experience has been that when the shaft and encoder are mated to within manufacturer-designated tolerances, our error function never exceeded the range limit error quoted by the manufacturer. The complete method of our encoder calibration is described in Ref. [68]. In this section, a short description of the calibration of both axes is given. Calibrations are also compared between two rebuilds of the instrument separated by a decade.

**Fig. 6:**
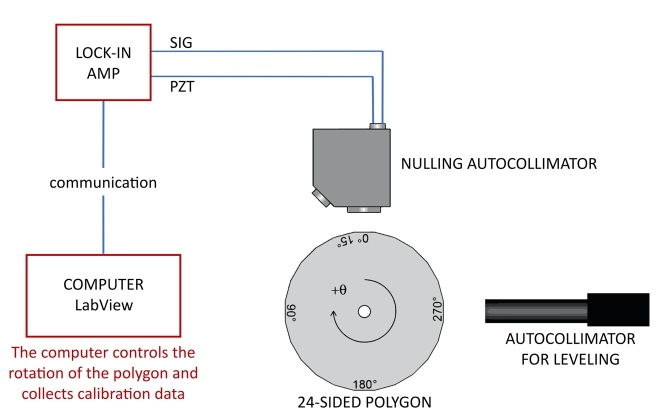
Overview of the method for the angle encoder calibration. The icosikaitetragon (24 sided polygon) is attached to the axis to be calibrated. Both a nulling autocollimator and an offset-measuring autocollimator are used. Inside the nulling autocollimator a rocking mirror is mounted on a piezoelectric stack powered by the lock-in amplifier (PZT). This moves the mirror about a central axis at a frequency determined by the lock-in amplifier reference signal (SIG).

**Fig. 7:**
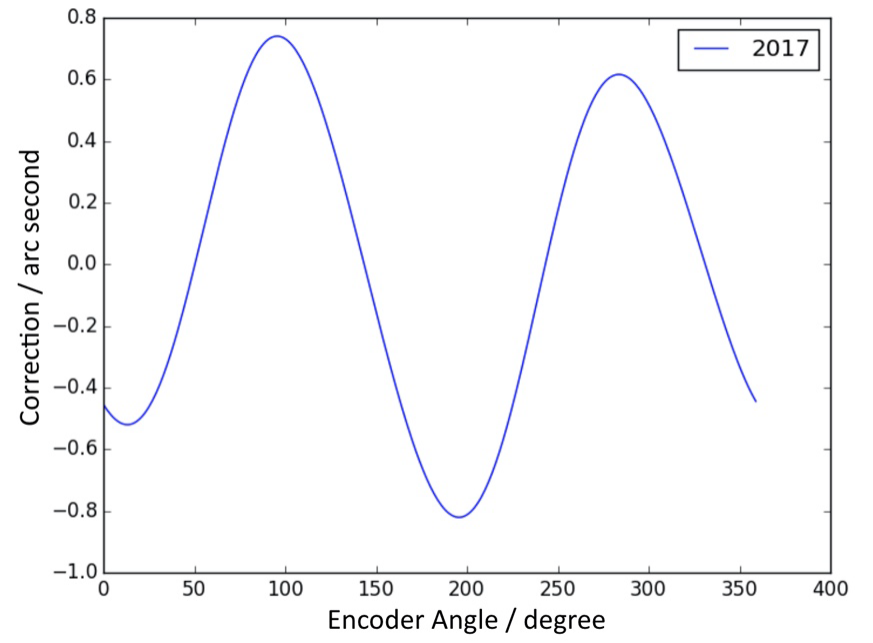
Result of encoder calibration, giving the correction function for axis A.

**Fig. 8:**
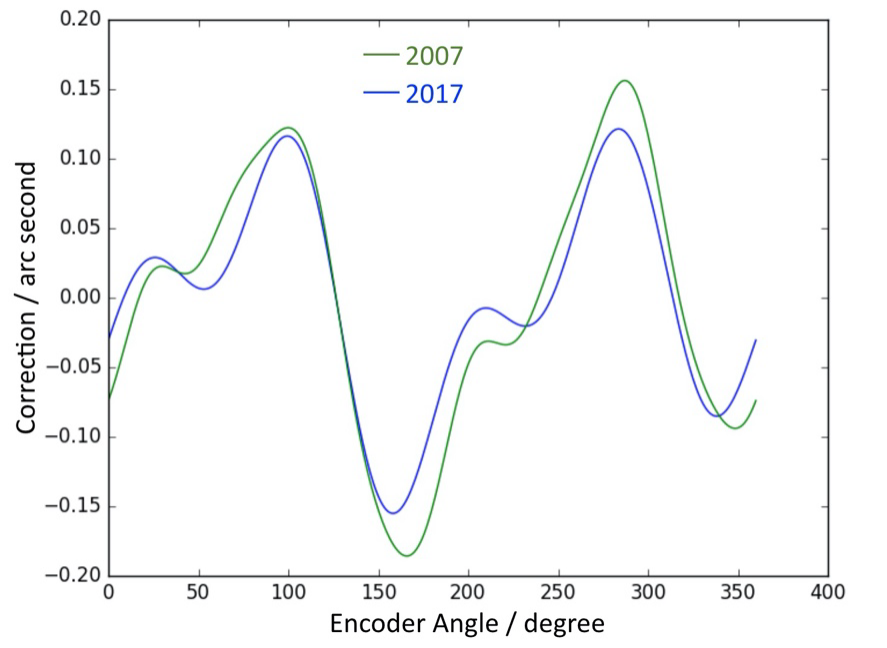
Correction function for the encoder on axis B. The calibration from 2007 is green, and the one from 2017 (current calibration) is blue.

An overview of the method for encoder calibration is shown in Fig. 6. The encoder calibration setup employs a twenty-four-sided mirrored artifact attached to the axis under calibration and monitored by an electronic nulling autocollimator. The artifact is a nearly regular icosikaitetragon with adjacent faces that differ from a nominal 15° by a few arc seconds. An offset-measuring autocollimator is used to measure the deviation of each polygon face from the mean face angle. The proper alignment of the polygon means that there are no sinusoidal variations to the face angles. The signal of the nulling autocollimator is collected with a lock-in amplifier that is read by the control computer. During the calibration, the rotation stage of the axis is stepped to turn between faces of the optical polygon, nulling on each face individually, to determine each face angle. The polygon is then rephased 23 times with the help of an external motor by 360/15 = 15°. Invoking circle closure, a least-squares system can be solved for both polygon face angles and encoder error function. Measurement campaigns are repeated in both clockwise and counterclockwise rotations to assess any torsional shaft windup or creep. The result is an uncertainty on a measured angle about 10 times smaller than the manufacturer’s specified (maximum) range error. Figures 7 and 8 show the correction functions for the encoders on axis A and axis B, respectively. Using these correction functions, the uncertainty in a single encoder reading is about 0.06 arc seconds [68]. This translates to a relative uncertainty Δ*E*/*E* = 0.2 × 10^−6^.

Figure 8 shows a previous calibration of the B axis conducted 10 years ago before the disassembly of the instrument in 2017. The similarity of the calibration curves reflects the sturdiness and reproducibility of the spectrometer construction and calibration technique.

### 2.8 Crystals and Crystal Mounts

**Fig. 9:**
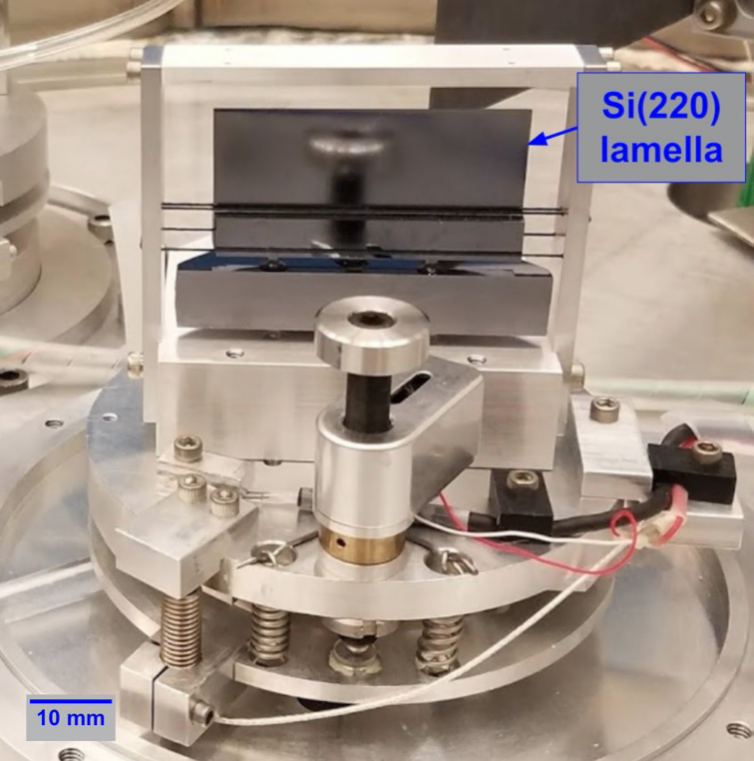
Crystal and crystal mount on axis A. The crystal’s diffracting surface is facing away from the viewer.

In the demonstration experiments that are highlighted below, silicon (220) crystals were mounted in both the A and B axis crystal holders. To facilitate alignment (Sec. 3.1), crystal A was a thin (≈ 0.45 mm) lamella with a thicker silicon base sitting directly on the adjustable support (Fig. 9). The active area of this lamella crystal was approximately 18 mm by 41 mm.

**Fig. 10:**
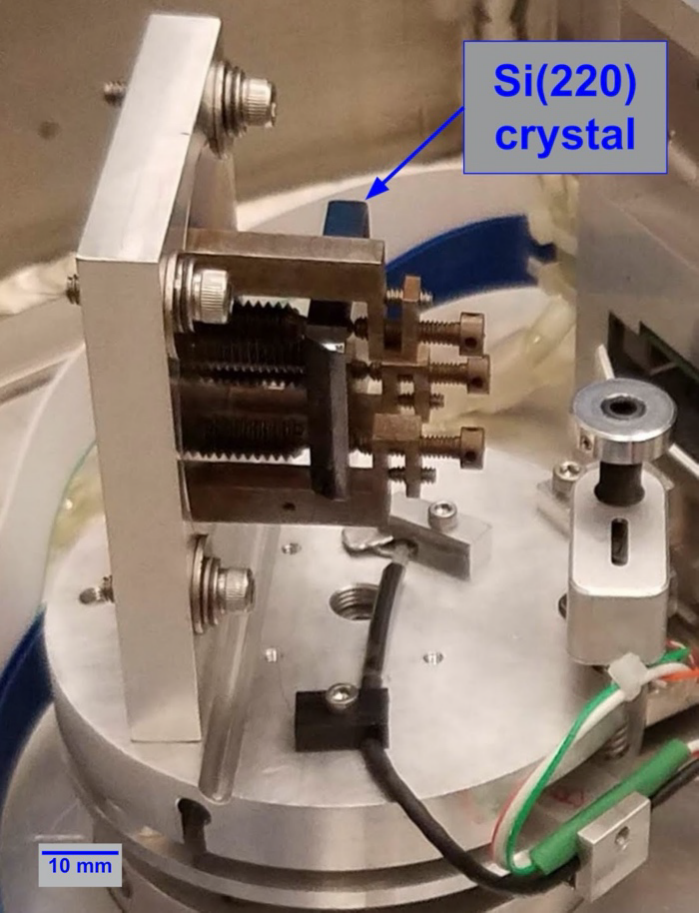
Crystal and crystal mount on axis B. The crystal’s diffracting surface is facing to the right in the picture.

The other crystal (B) was a 25 mm by 37.5 mm by 4 mm silicon piece sitting in a kinematic locator [69] originally developed for the VDCS instrument. A picture of the current crystal B setup is shown in Fig. 10. The design drawing of the crystal B holder can be seen on Fig. 3 of Ref. [1].

Both crystals were produced from ultrahigh-purity float-zone silicon boules. Their lattice spacing was determined by lattice spacing comparison measurements of samples from the same boule to a sample for which the lattice spacing had been determined by x-ray optical interferometry traceable to the SI definition of the meter. The lattice spacing measurements were described for both crystals in Ref. [70]. Crystal A of the VDCS originated from Wacker Siltronic and is designated WS1, while crystal B was cut from Wacker WS3-PBD. Table 1 shows the determined lattice spacings, *d*, for both crystals.

**Table 1:** Lattice spacings of Si(220) crystals utilized by VDCS as determined in Ref. [70].

Crystal	Year of NIST Lattice Comparison	*d*_Si(220)_ (*T* = 22.5 °C, *P* = 0 Pa)
B (WS1)	2016	192.015 565 8(52) × 10^−12^ m
A (WS3-PBD)	2013	192.015 571 2(52) × 10^−12^ m

The lattice spacing in both cases was determined with a relative uncertainty of Δ*d*/*d* = 2.71 × 10^−8^. The error bounds of both lattice spacing measurements overlapped with each other, and both were within range of the Committee on Data of the International Science Council (CODATA) 2018 value for *d*_220_ =192.015 571 6(32) × 10^−12^ m (in vacuum, 22.5 °C) from Table XXXIII of Ref. [71]. This was the value used for the analysis of all VDCS transition-energy measurements.

**Fig. 11:**
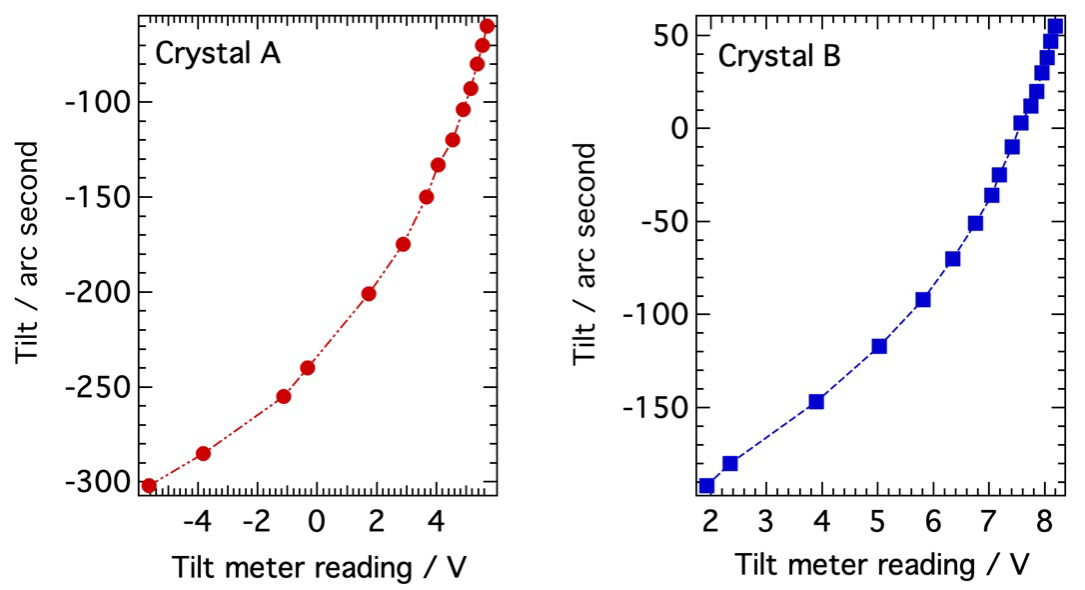
Tilt meter calibration curves used for setting crystal tilt during alignment.

The bases of the crystal mounts are identical and employ an aluminum plate with a flexure and a base ring with adjustment screws, one of which is a Picomotor™ for fine adjustment of the tilt of the crystals. The aluminum base plate has a long groove near the crystal, providing a flexure line such that when pressure is applied to the lever ring below the vertical plane, the crystal tilts. The Picomotor™, a New Focus 8302-UHV piezo motor actuator, is in tension with springs across the flexure opening, allowing control over the tilt of the crystals *in situ* during alignment with x rays. The tilt of each crystal is encoded with a MicroStrain NC-DVRT-1.5 induction proximity sensor which records the relative spatial separation (in volts) between the bracket lever arm and the base of the bracket. By recording the tilt meter readings while measuring the crystal tilt with a laser autocollimator, a calibration curve (Fig. 11) was produced for the tilt of each crystal out of the plane of rotation, and this curve was used during the alignment process described in Sec. 3.1.

The aluminum crystal holder base (on both axes) has a cylindrical extension that is notched and tightly forced onto the 25.4 mm shaft. It is secured to the shaft by a screw-tightened collar that has been upgraded from aluminum to steel. This more robust mounting was found to improve the stability of the crystal angle over time by about a factor of ten and assures the reproducibility of the peak position in the non-dispersive mode.

### 2.9 Detector

A critical upgrade of the VDCS was the introduction of a 2D x-ray detector. In the past, a flow proportional counter was used with a gas density stabilizer [39]. The performance of this detector was extended with the use of a shutter covering the upper or lower half of the detector. The intensity difference measured with either the top or the bottom half of the detector covered was used to improve the alignment of the optical path. The 2D x-ray detector allows for faster *in situ* crystal alignment and the quantification of the axial divergence correction in double-crystal instruments. Individual detector frames are used to derive transverse-integrated axial intensity (TIAI) plots, the production and use of which are demonstrated below in the Data Collection section (Sec. 3.1) and the Crystal Alignment section (Sec. 3.2).

The detector consists of a custom, vacuum-compatible version of the DECTRIS Pilatus 100K x-ray camera [72, 73]. This single-photon counting detector is based on the complementary metal–oxide–semiconductor (CMOS) hybrid pixel technology, where x rays are directly transformed into electric charge and processed in the CMOS readout chip. The 197 pixel by 487 pixel region on an area of 33.5 mm by 83.8 mm of a 450 μm thick silicon chip is bump bonded to the CMOS. The almost 100 000 square pixels measure 170 μm on a side. The detector can handle high count rates (10^7^ photons/s per pixel) and has been well demonstrated to operate even at low (1.57 keV) x-ray energies [74]. The nominal x-ray energy range of the NIST detector is 2.1 keV to 36 keV, with an adjustable low-energy discriminator of 1.6 keV to 18 keV. The energy resolution of the threshold is about 500 eV. Measured and calculated quantum efficiencies are in good agreement with each other according to Ref. [74]. For our measurements of copper *K* x rays, the efficiency correction was constant at 0.998 between the energies of 8000 eV and 8100 eV. Due to the large size of the incident beam, which spans close to a hundred pixels, we did not need a flatfield correction of the 2D detector.

**Fig. 12:**
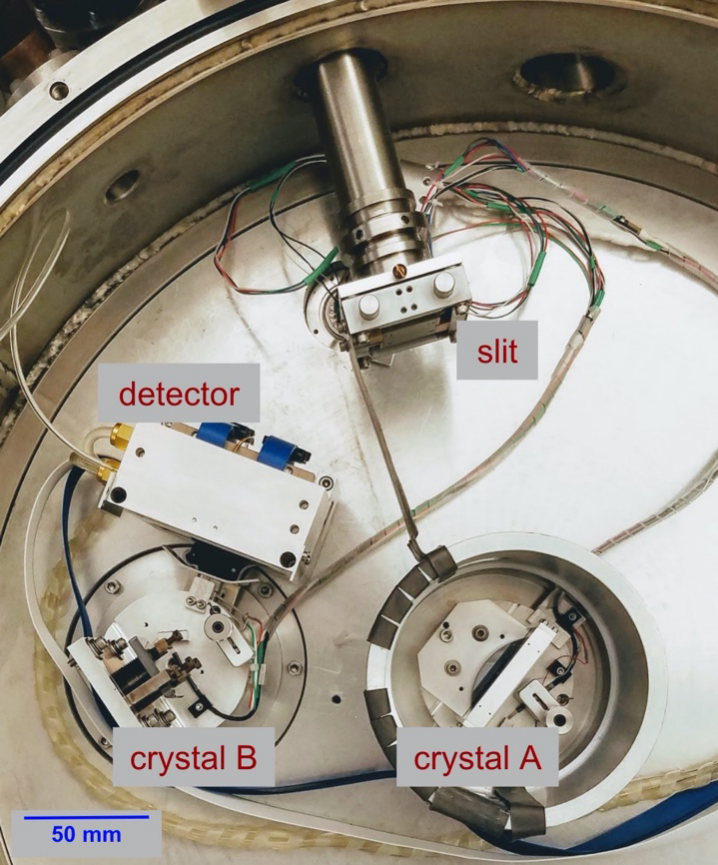
VDCS vacuum tank open from above showing the instrument in the dispersive mode. The detector cables and water lines can be observed running from the left side of the detector and permit detector motion counterclockwise around the crystal axis to the non-dispersive position. The port for the feedthroughs farther to the right from crystal A is not shown.

The DECTRIS camera head is water cooled, allowing the readout electronics to be operated at the desired ambient temperature in vacuum. Figure 12 consists of an overhead view of the interior of the vacuum tank, illustrating the source, diffraction crystals, and the detector. The power and readout cables can also be observed along with the water-cooling lines starting on the left side of the detector and wrapping around the chamber to the vacuum feedthrough port (not shown). This positioning allows free movement of the cables when changing the position of the detector from the dispersive to the non-dispersive modes. Figure 12 also shows the horizontal source slit that provides imaging of the source onto the camera in the vertical dimension.

## 3 Measurement and Analysis

The recently updated VDCS instrument was commissioned to confirm performance and to quantify all systematic corrections needed as well as their contributions to the uncertainty budget. This was done using the well-studied spectrum of Cu *Kα* [10].

### 3.1 Data Collection

**Fig. 13:**
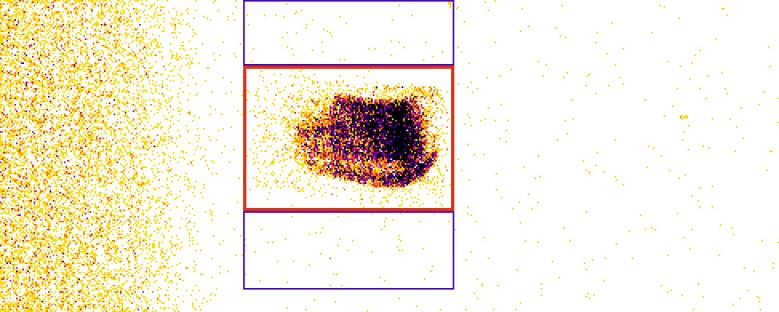
A single 10 s exposure x-ray image recorded with the area detector of the VDCS during a scan of crystal B. All 487 pixels by 195 pixels of the detector are shown. The data ROI is marked with a thick red rectangle, and the two background ROIs above and below the data ROI are marked in blue.

As described in the Principle of Operation section (Sec. 2.2), during data collection, crystal B is scanned while crystal A and the source are positioned to the angles corresponding roughly to the diffraction angle of the transition under investigation. The combined use of a horizontal slit and the 2D pixel detector facilitates a determination of the direct axial divergence correction of the recorded spectrum. At each step of the rotational scan of crystal B, an image is recorded by the area detector. During subsequent analysis, a region of interest (ROI) window is considered in which all diffracted x-ray photons from crystal B are included. Two “true background” ROIs are defined above and below the data region that integrate background counts during the measurements. The sum of the areas of the two background ROIs is the same as the area of the diffracted signal ROI. This area is used during data analysis for background subtraction. Figure 13 shows a single x-ray image from the full camera face indicating the data ROI (red rectangle) and the background ROIs (blue rectangles). This image was acquired with a 10 s exposure time and was taken during a dispersive scan of crystal B near the position of the Cu *Kα*_1_ peak. For the collection of these data, the (440) reflection was used. The left side of the image shows scattered x rays from spectrometer components that remains fairly constant during the dispersive scan. The extended source is vertically inverted due to the narrow slit (oriented within the plane of dispersion) and imaged horizontally by the angular acceptance of the diffraction crystals.

To create the spectrum, the total counts in the data ROI will be counted in each frame and stored as a function of the crystal B angle. The sum of all counts in the background ROIs will similarly be summed, stored, and fitted to then be subtracted from the stored data ROI counts. For detailed analysis, the counts of the data and background ROIs are treated separately, and a “smoothed” background will be subtracted from the data. The data ROI needs to be sufficiently large to include the whole image (all diffracted x-ray counts) during the entire scan. The x-ray beam will “walk” along the face of the x-ray detector as a function of energy, since the position has a slight energy dependence in the dispersive geometry. This walk of the image in the case of the Si(440) crystals is of the order of about 6 pixels on the detector between the Cu *Kα*_1_ and *Kα*_2_ peaks. For the case of the dispersive scans of the Cu *Kα* spectrum using Si(440) reflections, an angle range of 4° or 8° with 0.001° or 0.002° steps and 10 s or 5 s integration times at each step are employed. In the non-dispersive mode, where the narrow two-crystal rocking curve is recorded, the angular range of 0.006° is scanned in steps of 2 × 10^−5^ degrees. The width of the dispersive and non-dispersive diffraction curves for the system is of the order of 5 arc seconds.

### 3.2 Crystal Alignment

During alignment, the goal is to set the crystal faces parallel to each other and to their respective axes of rotation. The parallelism of the rotation axes was verified when the instrument was disassembled utilizing a Jewel LSRP Series inclinometer (Sec. 2.3). As observed in Fig. 4, the two axes are parallel to better than 6 arc seconds. In general, the angle offset due to crystal misalignment, Δ*θ*_B_, can be deduced from the axial divergence formula, as given in Eq. (24) of Ref. [10]:

$∆\theta_{B}=\frac{\psi^{2}}{2}\tan\theta_{B}+\left[\frac{\delta_{2}}{\cos\theta_{B}}+2\delta_{1}\sin\theta_{B}\tan\theta_{B}\right]\psi ,$ (2)

where $\theta_{B}$ is the Bragg angle, ψ is the angle offset of a given x-ray path from the horizontal, and $\delta_{1}$ and $\delta_{2}$ are the tilt angles of the two crystals out of vertical.

If we solve Eq. (2) for the extremum $∆\theta_{B0}=∆\theta_{B}\;(\psi_{0})$ for the so-called “zero row” position, we can get an estimate of the angle correction due to crystal misalignment.

$∆\theta_{B0}=\frac{-1}{2\tan\theta_{B}}{\left[\frac{\delta_{2}}{\cos\theta_{B}}+2\delta_{1}\sin\theta_{B}\tan\theta_{B}\right]}^{2}$ (3)

Since $∆\theta_{B0}$ is quadratic in $\delta_{1}$ and $\delta_{2}$, the crystal tilts do not strongly affect the measured peak position, so long as it is measured at its extremum. From Eq. (3), an angle offset, $∆\theta_{B0}$, producing a relative error Δ*E*/E ≈ 10^-6^ for the final result corresponds to tilts of *δ*_1_ = *δ*_2_ = 100 arc seconds. Based on this calculation, the crystal tilts shown in Fig. 4 are at least an order of magnitude smaller than a value that would affect the measurement accuracy within our tolerance.

**Fig. 14:**
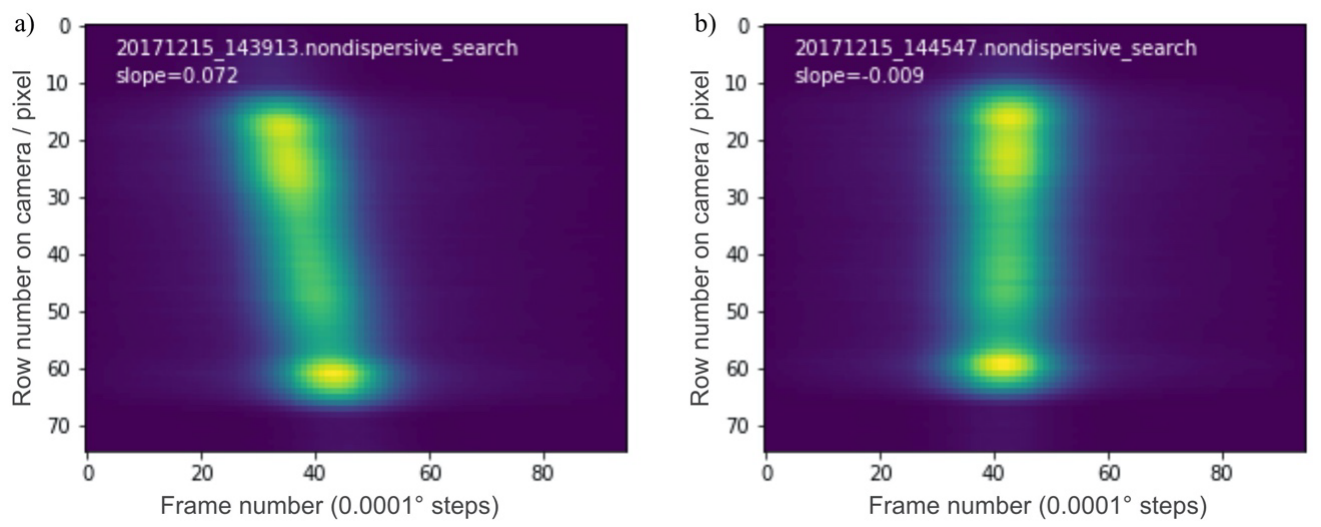
Composite images used in the alignment of the VDCS. The horizontal axis shows frame numbers, where each step in the scan through the non-dispersive diffraction condition was 0.0001°. At each step, the pixel values in the frame were row summed and plotted along the vertical axis. Slope is measured in arc seconds per pixel. (a) Misalignment between crystals A and B. (b) Aligned crystals (|slope| < 0.01 arc seconds/pixel).

The final alignment of the diffraction crystals is performed *in situ* with the crystals in vacuum and using x rays and the area detector. The spectrometer is set up in the non-dispersive mode, and crystal B is scanned through the diffraction condition using an angle step of (typically) 0.0001°. This scan produces a set of 2D x-ray images or frames that are used to produce a composite image, *viz*. the TIAI plot shown on Fig. 14. In this figure, the horizontal axis is frame numbers, where a non-dispersive scan was performed in 100 steps over a 0.01° angle range (0.0001° steps). At each angular position of the scan, we perform a row sum of pixel values on the frame recorded by the 2D detector. Each of these one-dimensional (1D) arrays is then displayed as a column in a composite image, the TIAI plot, where the column index is the crystal B rotation angle (or frame number). Hence, the individual frames, which look similar to Fig. 13 but vary in intensity, are used to produce TIAI plots such as those shown in Fig. 14. The tilted feature in Fig. 14(a) indicates a misalignment between the crystals.

Given that we can only measure a difference in tilt between the two crystals, an iterative procedure is followed that results in both crystals being aligned to the rotation axes of their respective stages. In the first part of the alignment procedure, we check crystal A (the lamella) by generating a TIAI plot such as that in Fig. 14(a). Crystal A is then rotated by 180°, and the tilt is checked again. With a few iterations and adjustments, when the two TIAI plots from either side of the lamella indicate an equal and opposite tilt, the lattice of crystal A is known to be parallel to its rotation axis. The tilt of crystal B is then adjusted to achieve parallelism with crystal A. The tilt of crystal B is adjusted until the feature on the composite TIAI plot is essentially vertical (slope is less than 0.01 arc seconds/pixel); see Fig. 14(b). This TIAI plot feature tells us that the two crystals’ diffraction planes are aligned with their rotation axes and are parallel to each other. As shown in Fig. 14(b), the slope is −0.009 arc seconds/pixel, which is equivalent to about 0.45 arc seconds overall misalignment over the 50 pixels of the feature measured vertically. This misalignment creates a negligible contribution to the uncertainty budget. Also, this procedure, in conjunction with the data of Fig. 4, demonstrates that crystal B will be in correct alignment when it is rotated into dispersive mode for collection of the actual spectra.

### 3.3 Diffraction Curves

**Fig. 15:**
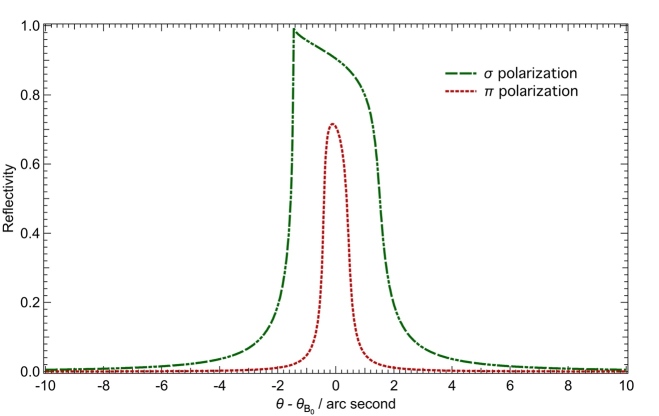
Calculated single-crystal rocking curves for the *σ* and *π* polarizations for the silicon 440 reflection at the Cu *Kα* x-ray energy (8048 eV).

The diffraction profiles for perfect silicon crystals can be calculated using dynamical diffraction theory [75]. We used the X-ray Oriented Programs package, XOP 2.4 [76] to calculate the dispersive and non-dispersive diffraction curves. First, the Darwin curves are calculated for the experimental diffraction planes, in this case, Si (440) in reflection. XOP’s Xcrystal 1.3 code calculates reflectivities for both *σ* and *π* polarizations with the option to center the curves at the (corrected) zero angle. Due to absorption, the Darwin–Prins curve in the *σ* polarization case has the well-known asymmetric shape at the Cu *Kα* x-ray energy (8048 eV) that is shown in Fig. 15.

**Fig. 16:**
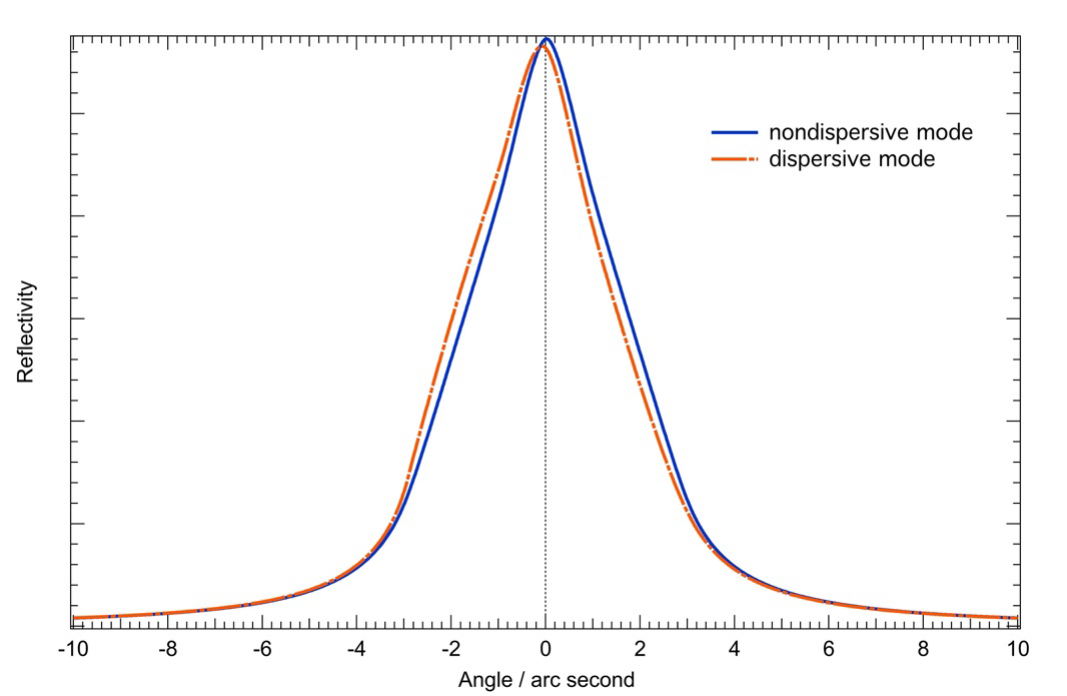
Calculated double crystal rocking curves in the non-dispersive and dispersive modes for the silicon 440 reflection plane at the Cu K*α* x-ray energy (8048 eV).

To produce double-crystal rocking curves, the *σ* and *π* polarized Darwin–Prins single-crystal curves are computed and convolved or autocorrelated with themselves (when identical crystals are used) to produce the dispersive and non-dispersive rocking curves, respectively. The *σ* and *π* polarizations are treated separately for the convolution or autocorrelation and are summed in the final step to produce the crystal diffraction curves. Figure 16 shows the calculated double-crystal rocking curves for the two VDCS modes. The rocking curve in the dispersive mode is slightly asymmetric and introduces a 0.1 arc seconds shift in the final data, which is accounted for in the analysis. The importance of this correction due to the asymmetry of the crystal response was pointed out by Chantler and Deslattes [77] in 1995.

**Fig. 17:**
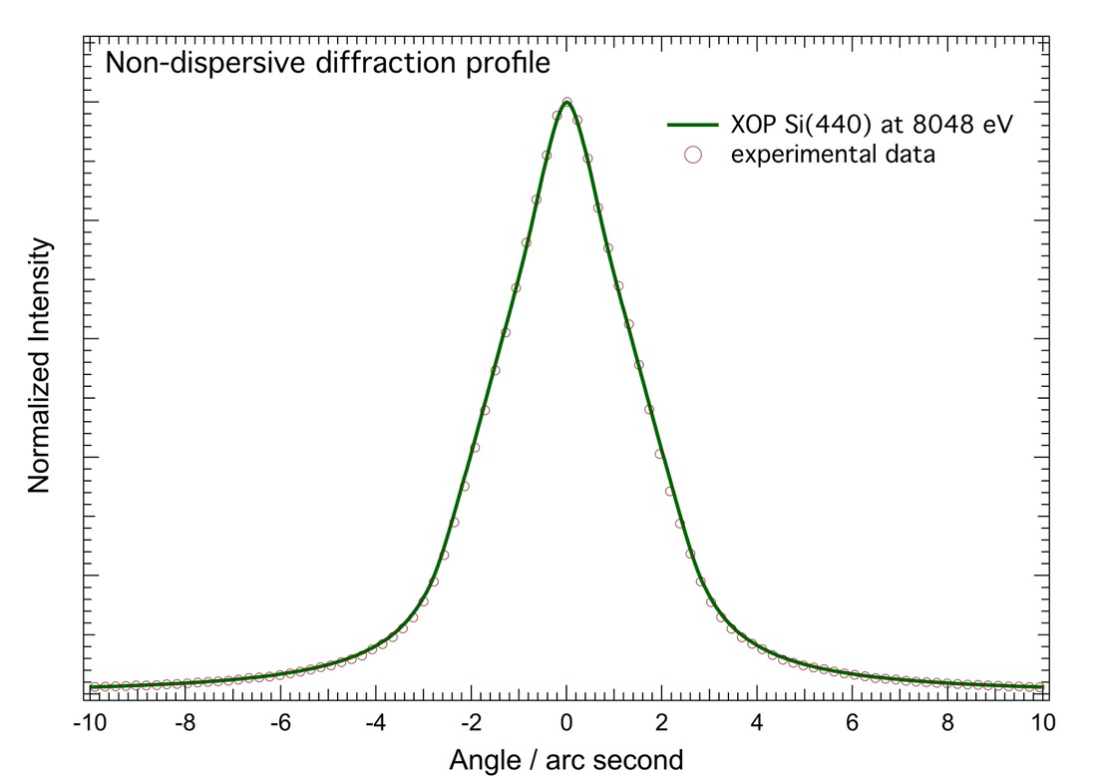
Experimental and calculated non-dispersive scans of Si(440) at 8048 eV. Error bounds for the measurement data points are within the size of the markers used on this graph.

The monochromatic rocking curve in the dispersive case is much narrower than the spectral features of the Cu *Kα* transition spectrum (the width of the Cu *Kα*_1_ peak is over 100 arc seconds), but in the non-dispersive geometry, the XOP calculation of the two-crystal rocking curve can be directly compared with the measurement, as shown in Fig. 17. We see excellent agreement between the experiment and XOP calculations, lending credibility to the corrections so determined.

#### 3.3.1 Stability of the Non-dispersive Peak

**Fig. 18:**
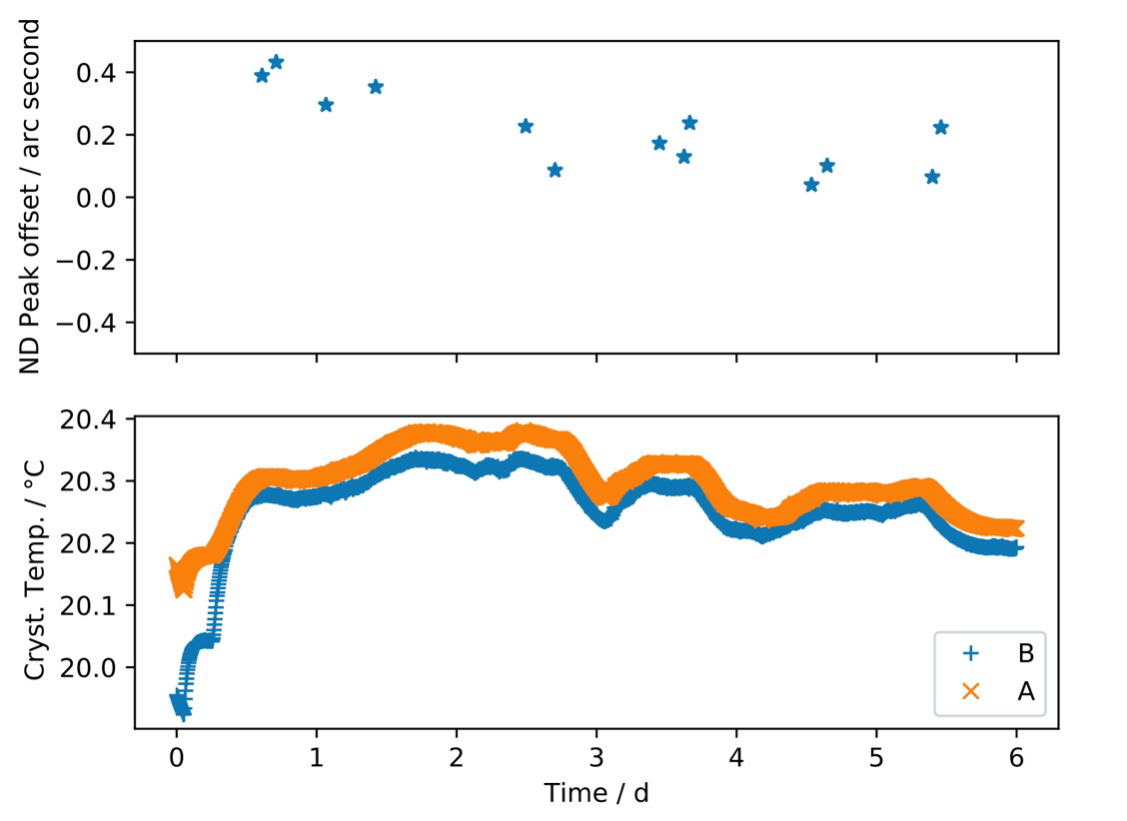
Non-dispersive (ND) peak position stability as a function of time. The non-dispersive peak offset in arc seconds is shown on the top panel. The bottom panel shows the readout of both crystal thermistors (A and B) during the time of the measurements.

The stability of the non-dispersive peak can serve as an indicator of the overall stability of the instrument. During the modernization of the instrument, we observed a temporal change of the position of the non-dispersive peak and initially found unexpected drifts (up to 10 arc seconds within hours). These observations led to the redesign of the crystal support attachment to the rotation shafts. The top panel of Fig. 18 shows the final long-term stability of the peak position in the non-dispersive mode with the redesigned crystal mounts. After a slight initial drift in the position, which was correlated with the stabilization of the system temperature, the non-dispersive peak remained stable within ± 0.1 arc seconds for several days, indicating that the modifications to the crystal mounts were effective in addressing the drift issue. This stability can be continuously verified during the measurements by collecting a non-dispersive scan after each dispersive scan.

### 3.4 Data Analysis

The analysis was performed as described in Refs. [10, 78]. Unlike in this previous work, the use of single bounce crystals in the case of the VDCS provides for a slightly different diffraction pattern with long tails instead of the triangular shape in the case of the channel-cut crystals used in the previous work [10, 78]. As observed in Fig. 17, the calculated theoretical curve matches the experiment. Another slight difference in the analysis is in the position of the area used for background on the 2D detector. In the previous work, the background was sampled from both sides (left/right) of the x-ray image (narrow source), while in the case of VDCS here, the background was sampled from the top and bottom of the image (extended source). During the analysis, the counts for each angle position were extracted from the raw x-ray images of the angle scans and corrected for the various systematics such as axial divergence, temperature, index of refraction, dynamical diffraction, and efficiency.

#### 3.4.1 Scan Parameters

The Cu *Kα* measurements were performed over the course of 4 d with continuous operation of the machine. Table 2 shows the instrument parameters used for these measurements. While each scan had 4000 steps, two used a smaller step size and a 10 s exposure time, and three used a larger step size and shorter exposure time to access a larger angle range.

**Table 2:** Data runs used for the measurement of the Cu *Kα* spectrum.

Index	*E*_min_ (eV)	*E*_max_ (eV)	Steps	Step Size in Degrees	Count Time (s)	Average Temperature (°C)	Emission Current (mA)
1	7996.4	8100.2	4000	0.001	10	20.17	50
2	7996.4	8100.2	4000	0.001	10	20.17	50
3	7946.4	8154.0	4000	0.002	5	20.20	50
4	7946.4	8154.0	4000	0.002	5	20.20	50
5	7946.4	8154.0	4000	0.002	5	20.22	50

#### 3.4.2 Axial Divergence Correction and Axial Fits

**Fig. 19:**
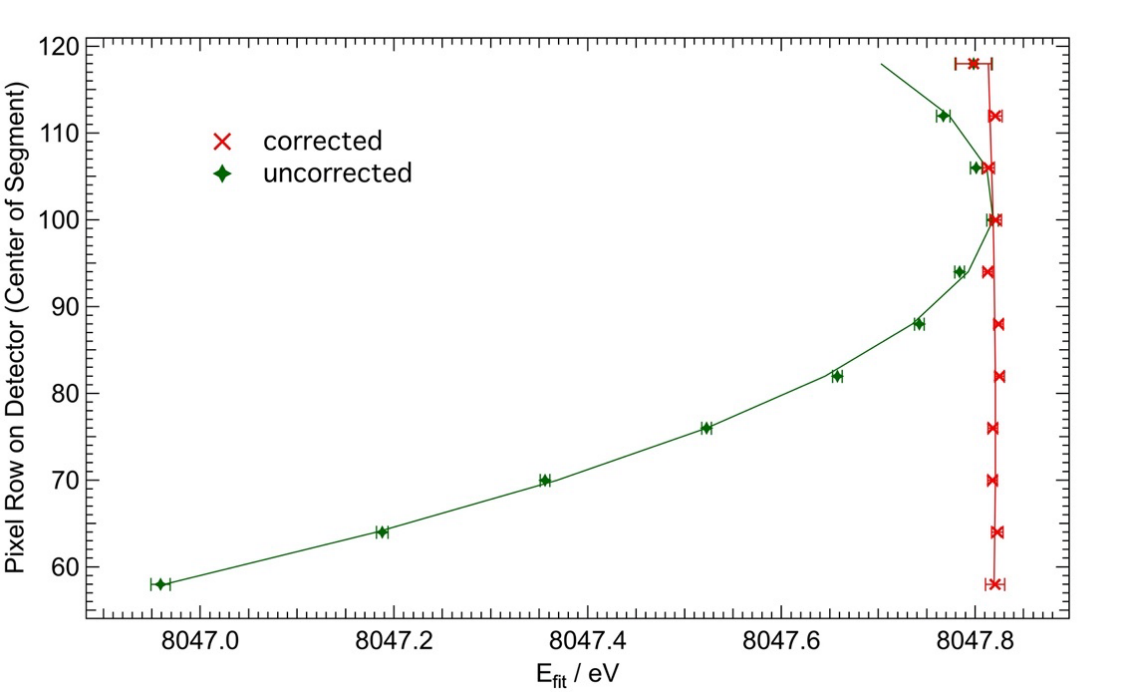
Apparent Cu *Kα*_1_ peak positions as a function of vertical row on the camera with a parabolic fit. The “corrected” set is chosen with *z*_0_ and *r*_0_ very close to optimum values. In the uncorrected case, *z*_0_ is off. Error bars are pure counting statistics (1σ).

A point source of x rays diffracting from a flat crystal will produce an arc pattern on a planar detector due to diffraction out of the plane of divergence. The feature of interest here is the diffraction angle associated with the extremum of this arc, which intersects the divergence plane. With the use of an extended source (Fig. 13), each point on the source potentially produces overlapping and displaced arcs on the detector. The use of a horizontal slit and an axial divergence correction permits the re-binning of out-of-plane photon counts to the correct diffraction angle, even for an irregularly shaped and extended source. Previously, in double-crystal instruments, the axial divergence correction (also called vertical divergence correction) had been a calculated offset in the final analysis based on a semi-empirical formula [66]. Our axial divergence correction was determined by the method described in Ref. [78]. To determine the optimal parameters for the axial divergence correction, the data collection region was divided into horizontal segments of 6 pixel rows on the 2D detector face. The data in each stripe were then fit to determine the peak positions for the Cu *Kα*_1_ peak of the Cu spectrum. An iterative fitting procedure was then used to find starting parameters of the axial divergence correction. The parameters of Table 3 were refined onto the peak positions to determine optimal values. Figure 19 shows a plot of these peak positions in the case of the correct *z*_0_ = 492 mm flight path parameter (in Fig. 19 labeled “corrected”) in red and an exaggerated *z*_0_ = 2000 mm flight path (labelled “uncorrected”) in green with *r*_0_ = 101 central row value.

**Table 3:** Parameters for the determination of the axial divergence correction.

Parameter	Description
*E*_0_	actual energy of the photons being measured
Δ*E*	energy correction due to axial divergence
Δ*y*	height of one row pixels in the x-ray detector
ψ	angle of the photon path from the perpendicular to the crystal axes (assuming crystals are aligned)
*r*_0_	row number on the camera, which is estimated to correspond to a photon path perpendicular to the crystal axes
*r*_1_	corrected central row number as a result of one pass of the calculation
*z*_0_	estimate of the distance from the slit to the x-ray detector
*z*_1_	corrected distance from the slit to the x-ray detector as a result of one pass of the calculation
*E*_max_	corrected energy for a ray measured at ψ = 0 (extremum of parabola)

This axial divergence correction method uses the imaging capability of the 2D detector with the help of a narrow slit, and after determination of the correct *z*_0_ and *r*_0_ parameters with the above fitting procedure (described in detail in Ref. [78]), it provides a corrected measurement spectrum and a calculated uncertainty for the axial divergence correction. This method works for extended sources that are irregular in shape and inhomogeneous in brightness.

#### 3.4.3 Efficiency Correction

Our efficiency correction considered wavelength-dependent corrections for self-absorption in the anode, detector efficiency, and crystal efficiency. The efficiency corrections were performed similar to those in Ref. [78], with silicon’s atomic scattering form factors *f*_1_ and *f*_2_ equivalent to those of Table 3 in Ref. [10]. The efficiency of the detector was constant across the energy range measured here. The calculated efficiencies from all three components summed to a negligible correction, with a relative uncertainty in the final calculated energy Δ*E*/*E* smaller than 0.01 × 10^−6^, and for this reason, they are not listed among the contributions to the type B uncertainty of the measurement (below).

#### 3.4.4 Fitting

**Fig. 20:**
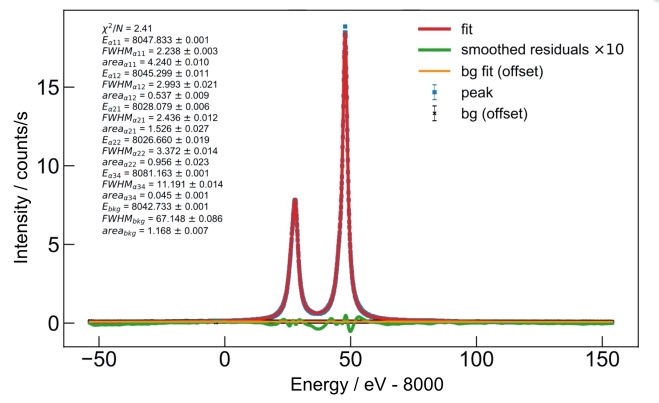
Data and fits for the Cu *Kα* spectrum acquired with the VDCS, where “bg” represents background, and FWHM is full width at half maximum.

To fit the spectra taken with the VDCS, similar procedures were used as those outlined in Refs. [10] and [78]. According to various authors [10, 32, 33, 79, 80], the optimal analytical profile shape function for fitting of the Cu *Kα* spectrum is a sum of four Lorentzian peaks. A fifth Lorentzian profile can be used to fit the less intense *Kα*_3,4_ complex on the high-energy shoulder of the *Kα*_1_ transition. The Cu *Kα* spectrum recorded with the VDCS and fit in such a manner is shown in Fig. 20. The five data sets listed in Table 2 were treated as one ensemble and were analyzed collectively for optimized fitting statistics.

The *Kα*_1_ and *Kα*_2_ peaks were fit with two Lorentzian peaks each, while the *Kα*_3,4_ peaks were handled as one unresolved feature. In order to get the fit to converge, an additional wide “background peak” was required. This additional artifact could be due to anisotropic fluorescence and scatter on spectrometer components produced by the extended source and/or unidentified background signals. According to the analysis, this additional background had no effect on the peak positions; nevertheless, we accounted for it in the error budget. Table 4 shows the fitting parameters with their ±1*σ* uncertainties.

**Table 4:** Peak parameters for the peaks fitted to the Cu *Kα* spectrum.

Component	Energy (eV)	±1*σ* of Energy	FWHM (eV)	±1*σ* of FWHM	Area (eV × counts/s)	±1*σ* of Area
*Kα*_11_	8047.833	0.001	2.238	0.003	4.240	0.010	
*Kα*_12_	8045.299	0.011	2.993	0.021	0.537	0.009	
*Kα*_21_	8028.079	0.006	2.436	0.012	1.526	0.027	
*Kα*_22_	8026.660	0.019	3.372	0.014	0.956	0.023	
*Kα* ^//a^	8081.163	0.001	11.191	0.014	0.045	0.001	
Background peak	8042.732	0.001	67.15	0.085	1.168	0.007	

^a^ Unresolved *Kα*_3,4_ 2*p* satellite structure.

### 3.5 Uncertainty Budget, Comparison, and Capability

Table 5 lists the corrections and the estimated uncertainties on those corrections in relative units. The uncertainties are dominated by angle errors (mainly limited by the non-dispersive peak stability), the uncertainties due to temperature, and the axial divergence correction.

**Table 5:** Contributions to the type B uncertainty.

Correction	Magnitude |Δ*E*/*E*| × 10^−6^	Uncertainty Δ*E*/*E* × 10^−6^	Explanation
Axial divergence	≈ 42	0.55	based on axial fit statistics
Slit height	0.01	0.05	Δ*θ* = (a^2^ + b^2^)/(24*L*^2^) tan *θ*
Temperature	2.35	0.60	*T_lab_* − (*T* = 22.5 °C) lattice spacing correction for the temperature difference from reference temperature; 0.2 °C temperature uncertainty
Index of refraction	11.78	0.02	*δ* / sin^2^ *θ*, limited by form factor uncertainty estimated at 0.2%
Dynamical asymmetry	0.35	0.01	asymmetry in diffraction curves
Angle errors	0.00	0.73	based on encoder calibration (*u_r_* = 0.2 × 10^−6^), axis and crystal misalignment (*u_r_* = negligible), and non-dispersive peak drifts up to 0.2 arc seconds between scans (*u_r_* = 0.7 × 10^−6^)
Si lattice spacing, *d*	0.00	0.03	lattice spacing measured to *u_r_* = 2.71 × 10^−8^ relative uncertainty [70]
Background	0.00	0.50	Background scatter skewing fit
**Quadrature sum**		**1.20**	

The total relative uncertainty *u_r_* = Δ*E*/*E* amounts to 1.2 × 10^−6^, similar to previous measurements [10, 33]. Table 6 shows a comparison of the peak top fit of the *Kα*_1_ peak with previously published results. The peak top position was computed by analytically differentiating the sum of the fitted Lorentzians and solving for the zero of the result. Columns 3 and 5 provide the relative differences that can be directly compared to the relative uncertainties of the measurement. Column 6 of Table 6 shows the calculated intensity ratio of the *Kα*_2_ and the *Kα*_1_ peaks, where the 0.52 value agrees with Mendenhall *et al.* [10].

**Table 6:** Comparison of the peak top position of Cu *Kα*_1_ with previous measurements: Mendenhall *et al.* [10] and Hölzer *et al.* [33]. Columns 3 and 5 provide the relative differences that can be directly compared to the relative uncertainties of the measurement.

	Peak Top (this work) / eV	Mendenhall *et al.* [10] / eV	Δ*E*/*E vs*. Mendenhall / 10^−6^	Hölzer *et al.* [33] / eV	Δ*E*/*E vs*. Hölzer / 10^−6^	*I*(*Kα*_2_)/*I*(*Kα*_1_) Ratio
*Kα*_1_	8047.8231(97)	8047.8162(10)	0.85	8047.8236(26)^a^	0.06	0.520

^a^ Value calculated with 2019 SI constants from wavelength.

## 4 Conclusion

The remodeled VDCS at NIST is now operational and ready to perform state-of-the-art quantum metrology with relative uncertainties of the order of Δ*E*/*E* = 10^−6^. The NIST VDCS is capable of providing improved low-energy (2 keV to 12 keV) x-ray wavelength/energy measurements on an SI-traceable scale. This and future measurements will provide a basis for further improvement of the standard list of x-ray transitions (*e.g.*, in SRD 128 [8]). In collaboration with other methods, *e.g*., with high-resolution energy-dispersive TES detectors, even faint x-ray features can be measured with unprecedented accuracy based on SI-traceable calibration scales provided by wavelength standards. Future measurements will include *L* transitions of rare earth metals to support interest in high-temperature superconductor research and remeasurements of many other *K* and *L* transitions that need verification and improvement on their uncertainties. Furthermore, the VDCS will target measurements of transitions with discrepancies between theory and measured results. The VDCS will also play a key role in providing standard reference data for a new x-ray spectral database as specified by the roadmap document of the international initiative on x-ray fundamental parameters [81].
